# Usefulness of positron emission tomography in the differentiation between tumor and infectious lesions in pediatric oncology: a case report

**DOI:** 10.1186/s12887-015-0427-3

**Published:** 2015-09-03

**Authors:** Fernanda Rodrigues Tibúrcio, Karla Emília de Sá Rodrigues, Hérika Martins Mendes Vasconcelos, Débora Marques Miranda, Ana Cristina Simões e Silva

**Affiliations:** Pediatric Oncology Service, Clinics Hospital, Federal University of Minas Gerais (UFMG), Belo Horizonte, Brazil; Center of Molecular Imaging, National Institute of Science and Technology of Molecular Medicine (INCT-MM), Belo Horizonte, MG Brazil; Department of Pediatrics, Interdisciplinary Laboratory of Medical Investigation, Faculty of Medicine, UFMG, Avenida Alfredo Balena, 190, 2nd floor, room# 281, CEP: 30130-100 Belo Horizonte, Minas Gerais Brazil

**Keywords:** Positron emission tomography, Tumor, Infection, Febrile neutropenia, Pediatric oncology

## Abstract

**Background:**

Sometimes, in pediatric oncology, it is difficult to differentiate the relapse of primary tumor from other diagnoses such as post-ischemic lesions or fungal abscess, without performing an organ biopsy. In addition, patients frequently are not under clinical conditions to be biopsied, mainly due to febrile neutropenia. A growing number of studies has focused on the use of Positron emission tomography/computed tomography with 18 Fluorodeoxyglucose ([^18^F]FDG-PET/CT) to distinguish tumor relapse from infectious lesions in patients with febrile neutropenia.

**Case presentation:**

This case report describes a 6 years-old girl with febrile neutropenia during the treatment of neuroblastoma. Blood culture showed Candida sp. Abdominal ultrasonography revealed multiple unspecific hypoechoic areas of variable sizes in spleen, which might be either tumor or Candida-induced abscesses. [^18^F]FDG-PET/CT was performed to help the diagnosis and revealed small splenic lesions highly suggestive of disseminated candidiasis. Patient was then treated with systemic antifungal agent. After the recovery from febrile neutropenia, a spleen biopsy was performed, confirming the diagnosis of fungal abscess. Due to the small size of lesions, modalities such as ultrasonography, CT and magnetic nuclear resonance were not able in distinguishing tumor relapse from infectious lesions.

**Conclusion:**

This case provides an excellent example in which the use of [^18^F]FDG-PET/CT is valuable in helping to localize potential sites of disseminated fungal infection to be diagnosed within clinical context. [^18^F]FDG-PET /CT seems to have a role in the evaluation of pediatric patients with febrile neutropenia.

## Background

Neuroblastoma is the most common pediatric extracranial tumor that arises from primitive neuroblasts of the embryonic neural crest. It can occur anywhere within the sympathetic nervous system, although the most common site of the primary tumor is in the abdomen [1, 2]. Neuroblastoma can metastasize by lymphatic and hematogenic dissemination to bone marrow, cortical bone, liver and skin [1].

**Fig. 1:**
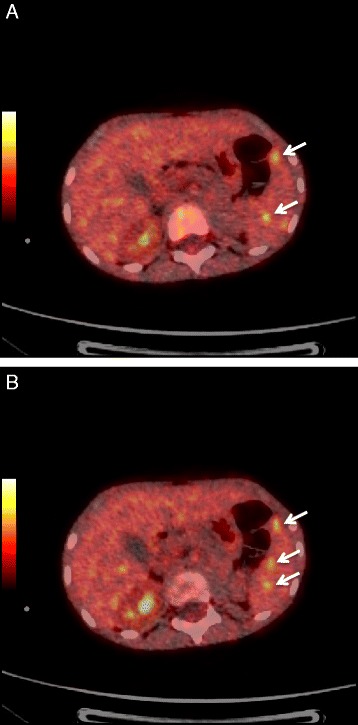
Panels (**a**) and (**b**) showed fused images of [^18^F]FDG-PET/CT (see white arrows) corresponding to focal hypermetabolic areas in spleen (highest was SUV_max_ = 3.2), previously visualized as hypointense images on computed tomography. Scanning was performed with 90 kV, 10–120 mAs (smart mA) and 3.75 mm section thickness. Acquisition time was 2 min per table position with 10-slice overlap at the FOV borders. PET image data sets were reconstructed iteratively (4 iterations and 24 subsets) by applying the CT data for attenuation correction. Fused images were displayed on a workstation using dedicated software

[^123^I]-MIBG scintigraphy is currently the tracer of choice for detection of neuroblastoma. It has high diagnostic accuracy and prognostic value for the assessment of patients after chemotherapy. Meticulous correlation with radiological examinations and recognition of the normal distribution pattern of [^123^I]-MIBG in children is vital to obtain optimal results [2]. For the evaluation of disease staging and therapeutic response, scans with [^123^I]-MIBG are routinely recommended. However, up to 10 % of patients may have tumors not avid for MIBG. In addition, even with recent advances on diagnostic tools and techniques, the differential diagnosis between disease relapse and infection can still be difficult [3]. With its technical superiorities, positron emission tomography/computed tomography ([^18^F]FDG-PET/CT) should be successfully introduced into the diagnostic workup of neuroblastoma [4].

[^18^F]FDG-PET/CT has shown an important role in managing pediatric patients with different malignancies. This technique has been considered a complementary diagnostic tool to conventional imaging methods [5]. [^18^F]FDG-PET/CT seems to be useful for the follow-up of asymptomatic treated patients and for the evaluation of symptomatic patients with suspected disease recurrence [5]. Early detection of residual tumors or lesions with small volume may optimize subsequent therapy. Limited data also suggest that [^18^F]FDG-PET/CT findings correlate well with histopathology and clinical outcome in children [6].

Timely identification of bloodstream infections due to spreading of the microorganisms to distant sites or metastatic complications of the main disease, although critical, is often difficult [7]. As [^18^F]FDG accumulates in activated leukocytes of infectious lesions, the use of [^18^F]FDG-PET/CT represents a promising imaging technique to detect these lesions in oncologic patients [8–10]. Proper staging and precise evaluation of residual lesions of invasive fungal infections are major challenges in the immune-compromised host. Preliminary data have suggested that [^18^F]FDG uptake may be observed in the course of active invasive fungal infections and it could be a helpful tool for the diagnosis [11]. The case reported here clearly shows the usefulness of [^18^F]FDG-PET/CT in the differentiation between tumor and infectious lesions in a febrile neutropenic pediatric patient.

## Case presentation

Our patient is a 6 years-old girl with relapsed adrenal neuroblastoma, INSS-stage 4, unfavorable histology and without information in regard to amplification of MYCN gene (MYCN-amplification). The [^123^I]-MIBG study showed abnormal radiotracer uptake in the left adrenal and liver. Neoadjuvant polychemotherapy with vincristine, etoposide, carboplatin and ifosfamide was effective and, after 6 courses, left adrenalectomy was performed. She was in complete remission after intense multimodal therapy. After 3 months receiving 13-cis-retinoic acid as post-consolidation chemotherapy, it was detected a hypodense mass on the left renal superior pole, which had not been observed in previous scans. The patient underwent a successful left nephrectomy and then a second-line cisplatin-based chemotherapy was started. After 3 courses, she presented febrile neutropenia and broad-spectrum antibiotics were prescribed. Regardless of the treatment, the patient exhibited persistent fever, not responsive to antibiotics, abdominal pain and hepatosplenomegaly. Preemptive amphotericin was then started and abdominal ultrasonography (US) was carried out. *Candida sp.* was identified in blood specimen culture. Abdominal US showed focal, nonhomogeneous, hypoechoic nodular splenic lesions, with diameter up to 2 cm, which might be either tumor or Candida-induced abscesses. At that point, [^18^F]FDG-PET/CT was performed since abdominal US and computed tomography (CT) failed to distinguish between tumor and infectious lesion.

Before [^18^F]FDG-PET/CT scanning, the patient was submitted to a fasting period of at least 6 h. Her blood glucose level (82 mg/dL or 4,5 mmol/L) was checked prior to intravenous injection of the radiopharmaceutical. The patient received an intravenous injection of 0,10 mCi/Kg of 2-deoxy-2-(^18^F)fluoro-D-glucose (^18^F-FDG), a glucose analogue. Data acquisition was performed by an integrated PET/CT system on a combined 64-slice CT/LYSO PET scanner (Discovery 690; GE Medical Systems, Milwaukee, WI, USA) within 50 min after injection. Data acquisition was obtained as follow: the patient was placed in a supine position on the scan with her arms raised above her head. A low dose CT scanning was performed first from the vertex to the feet, and, at this moment, scanning was performed with 90 kV, 10–120 mAs (smart mA) and 3.75 mm section thickness. Slice thickness was matched to PET section thickness. Immediately after CT scanning, a PET emission scan covering the same transverse field of view was obtained during normal breathing. Acquisition time was 2 min per table position with 10-slice overlap at the field of view (FOV) borders. PET image data sets were reconstructed iteratively (4 iterations and 24 subsets) by applying the CT data for attenuation correction. Fused images were displayed on a workstation using dedicated software. The [^18^F]FDG-PET/CT images were examined and interpreted by two experienced nuclear physicians and one radiologist. Based on the normal biodistribution of ^18^F-FDG, lesions were identified as foci with increased tracer and evaluated semi-quantitatively by the measure of maximum standardized uptake values corrected by lean body mass (SUV_max_). For comparison, a prior abdominal diagnostic CT scan was used.

The reconstructed PET images showed focal hypermetabolic areas in spleen (highest was SUV_max_ = 3.2), corresponding to the hypointense images previously detected in CT, suggesting the presence of inflammatory processes (Fig. 1). Although unlikely, we could not exclude the possibility of tumor lesions, due to previous diagnoses. Other PET findings were mild/moderate hypermetabolism in nostril and right maxillary sinus, indicating nasopharyngeal inflammatory process (Fig. 1). The images also showed the absence of the kidney and adrenal gland at left side, which were previously removed.

An increased pathological uptake of [^18^F]FDG was observed in all splenic areas previously identified by US. [^18^F]FDG-PET/CT revealed small splenic abscesses that were suggestive of disseminated candidiasis. Hence, it was very important to distinguish splenic lesions due to tumor from lesions caused by Candida infection. [^18^F]FDG-PET was used to determine the exact localization of the lesions and to establish the best surgical approach. She was further treated with splenectomy as she was a candidate for autologous bone marrow transplantation. Histological analysis of the spleen confirmed the diagnosis of candidiasis by showing only chronic inflammatory process without tumor cells. Nowadays, the child remains in complete remission 3 years after diagnosis.

## Conclusions

In this case, the US, which may allow the differentiation between neoplastic lesions, post-ischemic alterations or fungal abscess, was not conclusive. At this point, we had a diagnostic dilemma since the patient was still febrile with low platelet count, precluding the performance of splenic biopsy. Consequently, [^18^F]FDG-PET/CT scanning was recommended. This imaging technique *per se* suggested the infectious origin of spleen lesions and the absence of another focus of infection. This result allowed the proper treatment with antifungal agents with successful outcome.

This case clearly showed that, although [^18^F]FDG-PET/CT is not able to readily differentiate tumor versus inflammation, this technique is valuable in helping to localize potential sites of disseminated fungal infection to be diagnosed within clinical context and/or directed biopsy for further diagnosis [10–13]. Even if [^18^F]FDG-PET/CT is a state-of-the-art procedure for the diagnosis of multiple malignancies, it is still not a routine procedure for the differentiation between infection or malignancies due to high cost and limited availability.

[^18^F]FDG-PET/CT seems to be a valuable imaging method in helping the differentiation between tumor and infectious lesions. Therefore, [^18^F]FDG-PET/CT should be considered in challenging cases when the diagnosis is difficult, specially in pediatric patients with febrile neutropenia [12, 13].

## Consent

Written informed consent was obtained from the legal guardians and the patient to report this case and any accompanying images. A copy of the written consent is available upon request.

## Abbreviations

CT: Computed tomography
[^18^F]FDG: 18 Fluorodeoxyglucose
FOV: Field of view
MYCN: A gene on chromosome 2p24.3 that encodes a DNA-binding transcription factor. This gene is amplified in various tumours, especially in neuroblatomas
PET/CT: Positron emission tomography/computed tomography
[^123^I]-MIBG: 123Metaiodobenzylguanidine
US: Ultrasonography
